# Functional decline at the aging neuromuscular junction is associated with altered laminin-α4 expression

**DOI:** 10.18632/aging.101198

**Published:** 2017-03-14

**Authors:** Kah Meng Lee, Kirat K. Chand, Luke A. Hammond, Nickolas A. Lavidis, Peter G. Noakes

**Affiliations:** ^1^ School of Biomedical Sciences, The University of Queensland, St. Lucia, Queensland 4072, Australia; ^2^ University of Queensland Centre for Clinical Research, The University of Queensland, Herston, Queensland 4029, Australia; ^3^ Queensland Brain Institute, The University of Queensland, St. Lucia, Queensland 4072, Australia

**Keywords:** aging, laminin, maintenance, neuromuscular synapse, neurotransmission, remodeling

## Abstract

Laminin-α4 is involved in the alignment of active zones to postjunctional folds at the neuromuscular junction (NMJ). Prior study has implicated laminin-α4 in NMJ maintenance, with altered NMJ morphology observed in adult laminin-α4 deficient mice (*lama*4^−/−^). The present study further investigated the role of laminin-α4 in NMJ maintenance by functional characterization of transmission properties, morphological investigation of synaptic proteins including synaptic laminin-α4, and neuromotor behavioral testing. Results showed maintained perturbed transmission properties at *lama*4^−/−^ NMJs from adult (3 months) through to aged (18-22 months). Hind-limb grip force demonstrated similar trends as transmission properties, with maintained weaker grip force across age groups in *lama*4^−/−^. Interestingly, both transmission properties and hind-limb grip force in aged wild-types resembled those observed in adult *lama*4^−/−^. Most significantly, altered expression of laminin-α4 was noted at the wild-type NMJs prior to the observed decline in transmission properties, suggesting that altered laminin-α4 expression precedes the decline of neurotransmission in aging wild-types. These findings significantly support the role of laminin-α4 in maintenance of the NMJ during aging.

## INTRODUCTION

The neuromuscular junction (NMJ) is a highly specialized synapse allowing communication between the nerve terminal and the skeletal muscle fiber. Pre- and postsynaptic specializations must be aligned precisely for efficient neurotransmission [1-4]. The organization of these components is dependent on a group of signaling and adhesion proteins found localized at the basal lamina of the synaptic cleft, known as the laminins [5-9]. Laminins form heterotrimers that are made up of single α-, β- and γ-chains, of which laminin-221 (α2β2γ1), laminin-421 (α4β2γ1) and laminin-521 (α5β2γ1) form synapse specific laminin heterotrimers [6, 10, 11]. This study will focus in particular on the laminin-α4 chain found in laminin-421.

Laminin-α4 is found concentrated between the folds of the muscle fiber and it is suggested to play an instructive role in the placement of the active zones directly opposed to the postjunctional folds of the NMJ, with mice deficient in laminin-α4 (*lama*4^−/−^) displaying a higher proportion (~70%) of NMJs with misalignment of these specializations [12]. Further to this, studies conducted on 6 month old (6MO) *lama*4^−/−^ mice observed features of premature aging at the NMJs, such as fragmentation of acetylcholine receptors (AChRs), partial innervation and sprouting of axons, which became more evident with age [9]. These features are commonly observed at aging NMJs between 18-24MO in wild-type (WT) mice [13]. The same study found loss and mislocalization of laminin-α4 expression at the NMJs from 24MO WT mice. Together these findings indicate a potential role of laminin-α4 in the maintenance of normal and healthy adult NMJs [9]. Loss of NMJ integrity may be sufficient to initiate aging in skeletal muscle [14-16]. These changes are associated with decreased muscle mass and skeletal muscle weakness [17, 18], indicators commonly observed in aging humans and rodent models. It is therefore plausible that loss of laminin-α4 may be a significant contributor to muscle aging via the disruption in NMJ maintenance.

Morphologically, aging is thought to be accelerated at adult NMJs in the absence of laminin-α4, however the impact of these morphological alterations on neurotransmission is as yet unknown. It is also unclear as to whether the transmission properties at *lama*4^−/−^ NMJs match those observed at normal aging NMJs. The present study functionally compared neuro-transmission at adult and aged *lama*4^−/−^ NMJs to those of aged-matched WT NMJs. Additionally, we examined the neuromotor behavior of these *lama*4^−/−^ mice to investigate whether changes in transmission were reflected in production of hind-limb grip force. Finally, we tested whether changes in distribution and expression of laminin-α4 chain by immuno-histochemistry at WT NMJs was related to altered neurotransmission. Our results showed impaired transmission at *lama*4^−/−^ NMJs that remained consistent with increasing age. Aged WT NMJs (18-22MO) revealed similar transmission properties to that of *lama*4^−/−^ NMJs, which coincided with evident alterations in laminin-α4 expression. Similar trends were also observed in terms of the behavioral phenotypes, with *lama*4^−/−^ mice displaying weaker hind-limb grip force across age groups with WTs demonstrating an age-dependent decline. We observed alterations in the expression of synaptic proteins which supported our functional studies at *lama*4^−/−^ NMJs.

## RESULTS

### Altered neurotransmission properties remained constant at lama4^−/−^ neuromuscular junctions

**Figure 1:**
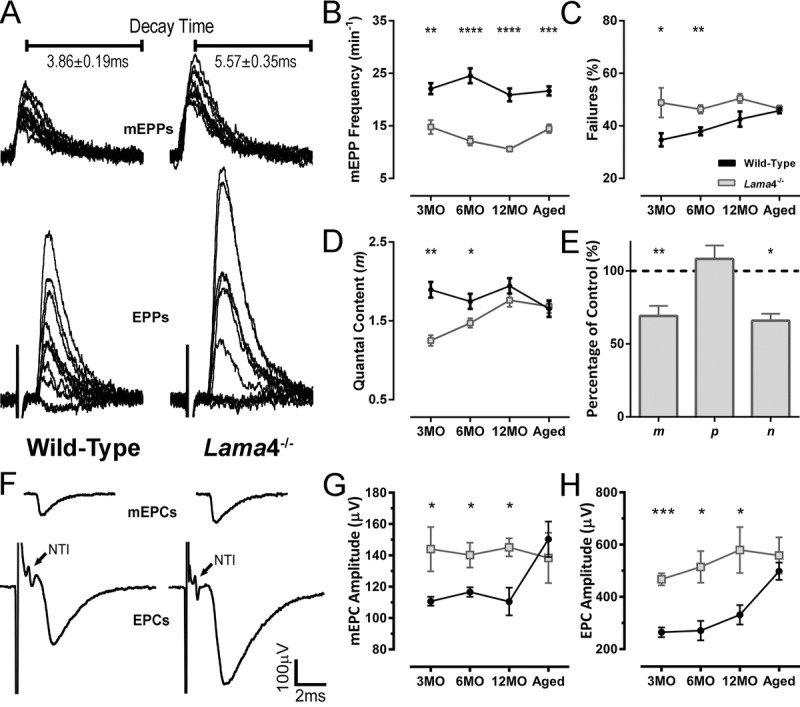
Altered transmission properties at *lama*4^−/−^ neuromuscular junctions were maintained throughout adulthood to aging (**A**) Sample traces from intracellular recordings of 10 consecutive mEPPs and 10 consecutive EPPs for WT and *lama*4^−/−^ NMJs at 3MO in diaphragm muscle. Prolonged mEPP decay time was observed as indicated on sample traces. Stimulus artefacts have been omitted from EPP traces for clarity. (**B**) mEPPs frequency, (**C**) failures in evoked release and (**D**) quantal content were measured across all ages investigated. (**E**) Normalized binomial parameter analysis displayed decreased quantal content (*m*) at 3MO *lama*4^−/−^ NMJs, which was resulted from lower number of active sites (*n*) with normal probability release of neurotransmitters (*p*). Binomial parameter analysis is normalized to 3MO WTs, as represented by dashed lines at 100%. (**F**) Sample traces from extracellular recordings of 10 averaged consecutive mEPCs and 10 averaged consecutive EPCs for WT and *lama*4^−/−^ NMJs at 3MO. (**G**, **H**) amplitudes of (**G**) spontaneous release (mEPCs) and (**H**) evoked release (EPCs) across all age groups. For all graphs, *n* = 4-6, NMJs = 25-45 per genotype at each age. Statistical analyses were performed using Student's *t*-test; values are presented as mean ± SEM (* *P* < 0.05, ** *P* < 0.01, *** *P* < 0.001 and **** *P* < 0.0001).

Prior study observed that *lama*4^−/−^ NMJs prematurely developed morphological features associated with aging which became more prevalent in an age-dependent manner [9]. Here we asked how the higher prevalence of these features affects neurotransmission with age. *Lama*4^−/−^ NMJs at 3MO displayed larger amplitudes of spontaneous release (miniature endplate potentials; mEPPs) and evoked release (endplate potential; EPPs) (Fig. 1A). Timing parameters such as mEPP decay time were significantly longer at 3MO *lama*4^−/−^ NMJs (*P* = 0.0027; 5.57 ± 0.35 ms) when compared with 3MO WT (3.86 ± 0.19 ms; Fig. 1A). The longer mEPP decay time at *lama*4^−/−^ NMJs was maintained across the other ages investigated when compared with WT NMJs (6MO: *P <* 0.0001; WT; 4.13 ± 0.15 ms *vs. lama*4^-/^; 5.77 ± 0.15 ms*,* 12MO : *P <* 0.0001; WT; 4.30 ± 0.17 ms *vs. lama*4^-/^; 6.01 ± 0.18 ms, Aged: *P <* 0.0001; WT; 4.03 ± 0.07 ms *vs. lama*4^-/^; 5.01 ± 0.09 ms). Frequency of spontaneous release was also significantly reduced and persisted similarly at all ages investigated for *lama*4^−/−^ NMJs in comparison with WT NMJs (3MO: *P* = 0.003; WT; 22.08 ± 1.04 min^-1^ *vs. lama*4^−/−^; 14.78 ± 1.31 min^-1^, 6MO: *P <* 0.0001; WT; 24.54 ± 1.41 min^-1^ *vs. lama*4^-/^; 12.13 ± 0.87 min^-1^*,* 12MO: *P <* 0.0001; WT; 20.91 ± 1.23 min^-1^ *vs. lama*4^-/^; 10.60 ± 0.45 min^-1^*,* Aged: *P =* 0.0005; WT; 21.65 ± 0.87 min^-1^ *vs. lama*4^-/^; 14.47 ± 0.81 min^-1^; Fig. 1B).

*Lama*4^−/−^ NMJs displayed a higher rate of failed EPP responses, which may be due to intermittent failure in propagation of the nerve action potential or in depolarization-secretion coupling at the NMJ itself. This increased failure in evoked responses remained unchanged across age groups, when compared with WT NMJs (3MO: *P =* 0.0425; WT; 34.69 ± 2.52% *vs. lama*4^-/^; 48.84 ± 5.64% and 6MO: *P =* 0.0044; WT; 37.90 ± 1.50% *vs. lama*4^-/^; 46.32 ± 1.61%; Fig. 1C). The rate of failed EPP responses gradually increased with age at WT NMJs until 12MO, where no significant difference was observed when compared with age-matched *lama*4^−/−^ NMJs (*P =* 0.0544; WT; 42.58 ± 2.89% *vs. lama*4^−/−^; 50.46 ± 1.75%; Fig. 1C). At aged NMJs, WTs (45.78 ± 0.96%) reached a similar EPP failure rate to that of *lama*4^−/−^ (*P* = 0.6197; 46.56 ± 1.16%; Fig. 1C).

Quantal content was significantly different between both genotypes at 3MO (*P =* 0.0015; WT; 1.90 ± 0.10 *vs. lama*4^−/−^; 1.25 ± 0.07) and 6MO (*P* = 0.0314; WT; 1.75 ± 0.10 *vs. lama*4^−/−^; 1.47 ± 0.06; Fig. 1D). At 12MO and aged NMJs, no significant difference in quantal content was found between WT and *lama*4^−/−^ NMJs (12MO: *P* = 0.1995; WT; 1.94 ± 0.10 *vs. lama*4^−/−^; 1.76 ± 0.08 and Aged: *P* = 0.8287; WT; 1.65 ± 0.10 *vs. lama*4^−/−^; 1.68 ± 0.08; Fig. 1D). Based on normalized binomial parameter analysis, the lower quantal content at 3MO *lama*4^−/−^ NMJs (69.1 ± 6.89% of WT, *P* = 0.01) occurred as a result of a decrease in the number of active release sites (*P* = 0.0227; 65.92 ± 4.69%), with normal probability of transmitter release (*P =* 0.5196; 108.3 ± 9.16%; Fig. 1E).

We next investigated the propagation of the action potential using extracellular recordings, which allowed examination of the nerve terminal impulse (NTI) in order to determine whether this may be a contributing factor to the higher failures in evoked responses seen at *lama*4^−/−^ NMJs. Based on our findings, NTIs were present and remained consistent throughout the recordings in *lama*4^−/−^ at 3MO (Fig. 1F, arrows), which was observed at all ages investigated. These findings suggest there was no issue with action potential propagation, but rather a defect at the NMJ itself as the cause of the higher failure rate of evoked release at *lama*4^−/−^ NMJs.

At 3MO, *lama*4^−/−^ displayed larger amplitudes of miniature endplate currents (mEPCs) and endplate currents (EPCs) compared with WT (Fig. 1F). Spontaneous amplitude remained constant at 3MO (144.1 ± 14.1 μV), 6MO (140.2 ± 8.0 μV), 12MO (145.2 ± 5.9 μV) and aged group (138.4 ± 16.0 μV) at *lama*4^−/−^ NMJs (*P* = 0.9758; one way ANOVA).

Spontaneous amplitude was significantly higher at *lama*4^−/−^ NMJs when compared with age-matched WTs (3MO: *P* = 0.0496; 110.7 ± 2.8 μV, 6MO: *P* = 0.0396; 116.6 ± 3.1 μV and 12MO: *P* = 0.0111; 110.5 ± 8.8 μV; Student's *t*-test, Fig. 1G). Of note, aged WT NMJs (150.3 ± 11.3 μV) increased significantly from values observed at 12MO WTs by 36.02% (*P* = 0.0106; one way ANOVA), with no significant difference found when compared with aged *lama*4^−/−^ NMJs (*P* = 0.5718; 138.4 ± 16.0 μV; Fig. 1G). Similar trends were also observed for EPC amplitudes at *lama*4^−/−^ NMJs across age groups when compared with age-matched WT NMJs (3MO: *P* = 0.0001; WT; 264.5 ± 18.4 μV *vs. lama*4^−/−^; 467.2 ± 23.1 μV, 6MO: *P* = 0.0172; WT; 270.9 ± 37.0 μV *vs. lama*4^−/−^; 514.7 ± 60.9 μV and 12MO: *P* = 0.0318; WT; 331.4 ± 37.4 μV *vs. lama*4^−/−^; 579.6 ± 87.9 μV; Student's *t*-test, Fig. 1H). Investigation of aged NMJs revealed similar EPC amplitude size between both genotypes (*P* = 0.4806; WT; 491.8 ± 33.0 μV *vs. lama*4^−/−^; 558.6 ± 69.3 μV; Fig. 1H) as aged WT increased in EPC amplitude significantly from 12MO WT (*P =* 0.0089; one way ANOVA). *Lama*4^−/−^ NMJs on the other hand, consistently displayed similar EPC amplitude size across different ages (*P* = 0.3988; one way ANOVA).

### Lower density of active zones at *lama*4^−/−^ neuromuscular junctions from adulthood to aging

**Figure 2:**
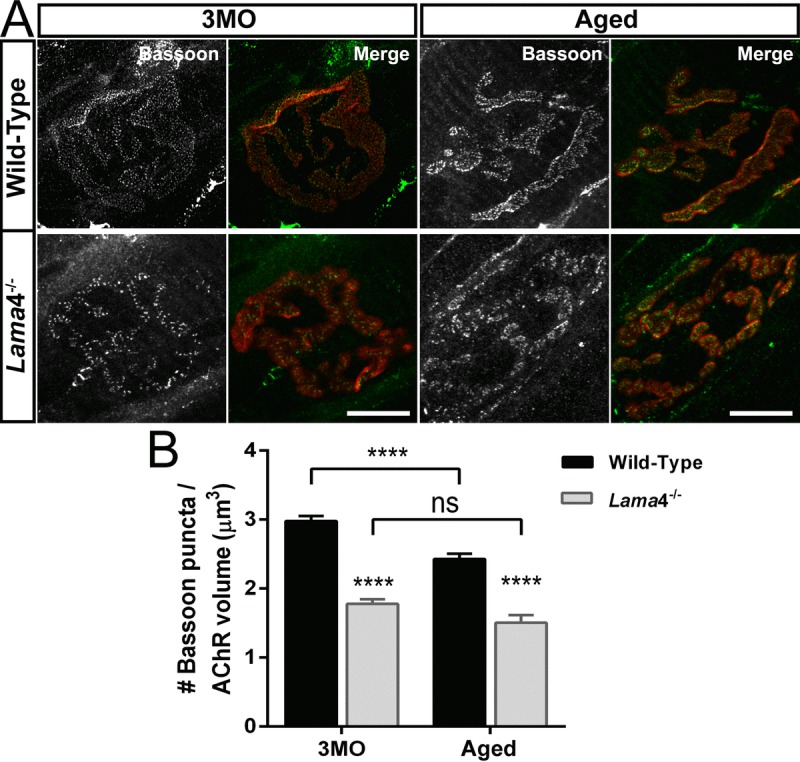
Decreased density of active zone marker Bassoon at *lama*4^−/−^ neuromuscular junctions during adulthood and aging (**A**) Representative staining of Bassoon (grayscale in single channel and green in merged channels) with respect to postsynaptic AChR endplate (red) in diaphragm muscle. WT NMJs at both ages; 3MO and aged group presented uniformly distributed Bassoon puncta while aged-matched *lama*4^−/−^ NMJs showed irregular distribution of Bassoon puncta. Scale bar = 10 μm. (**B**) Bassoon density measured by number of puncta over volume of postsynaptic AChR endplates. While aged WT NMJs maintained uniform pattern of Bassoon puncta, the measurement of Bassoon density clearly showed decreased density in comparison with 3MO WT. *Lama*4^−/−^ NMJs on the other hand, consistently showed decreased Bassoon density from 3MO to aged group. *n* = 3 for each genotype per age, NMJs = 47-55 for WT, NMJs = 33-69 for *lama*4^−/−^. Statistical analysis was performed using two-way ANOVA with Tukey's post hoc test; values are presented as mean ± SEM (**** *P* < 0.0001).

Our functional findings indicated a decrease in the number of active release sites; we therefore next investigated if there was a change in active zones distribution using Bassoon. At 3MO, WT NMJ showed uniform distribution of Bassoon puncta colocalized to the postsynaptic endplate whereas aged-matched *lama*4^−/−^ NMJ displayed irregular distribution of Bassoon puncta (Fig. 2A). Quantification of expression showed a 40.32% (*P* < 0.0001) higher density at WT NMJs (2.98 ± 0.07 puncta/μm^3^) compared with *lama*4^−/−^ NMJs (1.78 ± 0.07 puncta/μm^3^; Fig. 2B). Aged WT NMJs displayed a decrease in Bassoon density by approximately 18.46% from that observed at 3MO WT NMJs (*P* < 0.0001; Aged: 2.43 ± 0.08 puncta/μm^3^ *vs*. 3MO: 2.98 ± 0.07 puncta/μm^3^; Fig. 2A and B). Despite the drop in Bassoon density, aged WT Bassoon density remained significantly higher (*P* < 0.0001) than that of aged *lama*4^−/−^ (1.54 ± 0.11 puncta/μm^3^; Fig. 2B). The density of Bassoon at *lama*4^−/−^ NMJs remained consistently low showing no age-dependent decline (*P* = 0.0978; Fig. 2B), a finding similar to the trends observed with our functional investigation of neurotransmission at *lama*4^−/−^ NMJs (Fig. 1B-C, G-H).

### Weakening of hind-limb grip force in *lama*4^−/−^

**Figure 3:**
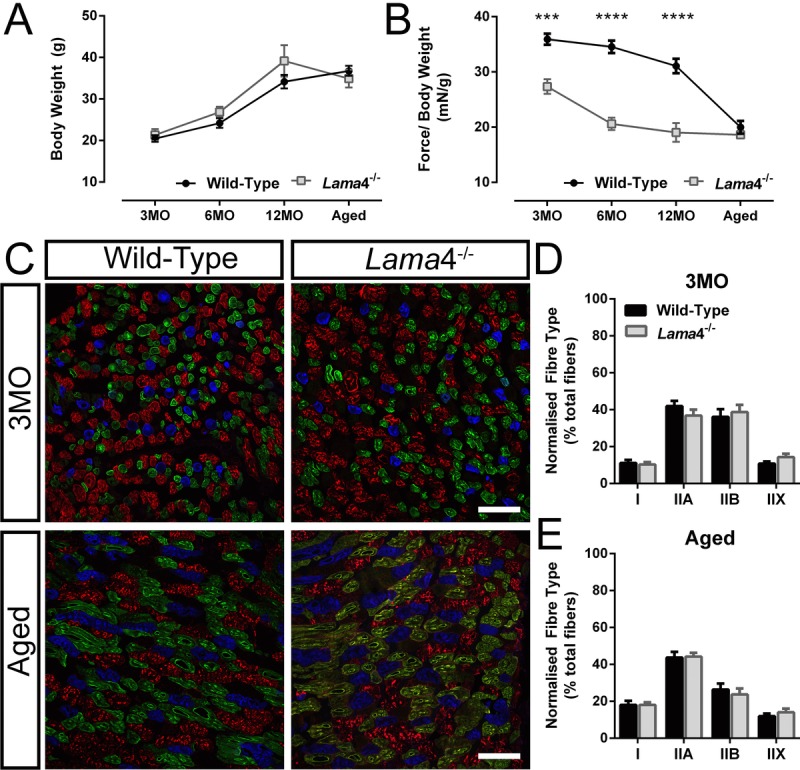
Weaker hind-limb grip force but normal distribution of different fiber types in *lama*4^−/−^ (**A**) Body weight of animals and (**B**) normalized grip force over body weight. Aged WT dropped in grip force/body weight tremendously and reached the similar level of motor performance to that of *lama*4^−/−^. (**C**) Triple labelling staining for different fiber types at 3MO and aged group; blue staining represents Type I, green represents Type IIA, red represents Type IIB and unstained fibers represents Type IIX in gastrocnemius muscle. Scale bar = 100 μm. (**D**, **E**) Distribution of each different fiber types at both ages of 3MO and aged was normal in both genotypes. (**A**-**B**), *n* = 8 for each genotype per age group investigated. (**D**-**E**), *n =* 5 for each genotype*,* with >1000 fibers counted for each genotype at 3MO and aged groups. Statistical analyses were performed using Student's *t*-test; values are presented as mean ± SEM (*** *P* < 0.001 and **** *P* < 0.0001).

Previous study observed uncoordinated hind-limb movement in *lama*4^−/−^ mice suggestive of neuromuscular dysfunction [12]. We further explored perturbations in neuromuscular function through assessment of hind-limb grip force in order to investigate the link between impaired transmission and muscle function. No significant difference in body weight was observed across age groups (Fig. 3A). Normalization of peak hind-limb grip force to body weight revealed significantly weaker force production in *lama*4^−/−^ mice compared with WTs at 3MO, 6MO and 12MO (3MO: *P* = 0.0002; WT; 35.92 ± 1.01 mN/g *vs. lama*4^−/−^; 27.35 ± 1.34 mN/g, 6MO: *P* < 0.0001; WT; 34.54 ± 1.13 mN/g *vs. lama*4^−/−^; 20.59 ± 1.11 mN/g and 12MO: *P* < 0.0001; WT; 31.07 ± 1.30 mN/g *vs. lama*4^−/−^; 19.04 ± 1.69 mN/g; Fig. 3B). Aged WTs (19.97 ± 1.15 mN/g) declined significantly (*P* < 0.0001; one way ANOVA) from 12MO WT by 35.73% with no significant difference found when compared with aged *lama*4^−/−^ (*P* = 0.3323; 18.63 ± 0.68 mN/g; Fig. 3B).

The weaker hind-limb grip force in *lama*4^−/−^ may be associated with changes in fiber type composition or disrupted neurotransmission at the NMJs. In order to determine this, we stained for expression of different myosin fiber types in the gastrocnemius muscle, as this muscle has been shown to be involved in the production of hind-limb grip force [19-21]. No differences were found between WT and *lama*4^−/−^ at 3MO for each fiber types; type I (*P* = 0.6845; WT; 11.16 ± 1.60% *vs. lama*4^−/−^; 10.26 ± 1.40%), type IIA (*P* = 0.2701; WT; 41.93 ± 2.89% *vs. lama*4^−/−^; 36.76 ± 3.26%), type IIB (*P* = 0.6623; WT; 36.11 ± 4.19% *vs. lama*4^−/−^; 38.71 ± 3.91%) and type IIX (*P* = 0.1480; WT; 10.80 ± 1.12% *vs. lama*4^−/−^; 14.26 ± 1.85%; Fig. 3C and D). Type I fibers in aged muscles increased significantly in both WT (*P =* 0.0363; 18.04 ± 2.22%) and *lama*4^−/−^ (*P* = 0.0042; 18.06 ± 1.39%) when compared with 3MO WT (11.16 ± 1.60%) and 3MO *lama*4^−/−^ (10.26 ± 1.40%) respectively (compare Fig. 3D with E). Aged WT (26.35 ± 3.29%) had a non-significant decrease in type IIB fibers from 3MO WT (*P* = 0.1041; 36.11 ± 4.19%; compare Fig. 3D with E). Aged *lama*4^−/−^ NMJs had a significant decrease in type IIB fibers from 3MO *lama*4^−/−^ by 38.70% (*P* = 0.0179; compare Fig. 3D with E). However, no changes were found when comparing each different fiber types between aged WTs and *lama*4^−/−^ (Type I: *P* = 0.9935; WT; 18.04 ± 2.22% *vs. lama*4^−/−^; 18.06 ± 1.39%, Type IIA: *P* = 0.8972; WT; 43.68 ± 3.11% *vs. lama*4^−/−^; 44.18 ± 2.12%, Type IIB: *P* = 0.5832; WT; 26.35 ± 3.29% *vs. lama*4^−/−^; 23.73 ± 3.19%, and Type IIX: *P* = 0.4209; WT; 11.94 ± 1.42% *vs. lama*4^−/−^; 14.03 ± 2.02%; Fig. 3C and E).

### Reduced facilitation and increased depression in transmitter release during high frequency stimulation are associated with lower density of vesicles at *lama*4^−/−^ neuromuscular junctions

Our findings showed that weakening of hind-limb grip force in *lama*4^−/−^ are not a result of altered fiber type distribution, suggesting that transmission in hind-limb muscles of *lama*4^−/−^ may be responsible for the weaker grip force observed in mutants. In order to investigate these changes we used intracellular electrophysiology to record transmission in the hind-limb *extensor digitorum longus* (EDL), another muscle which is also involved in production of hind-limb grip force [22], under physiological levels of extracellular calcium (2 mM).

**Figure 4:**
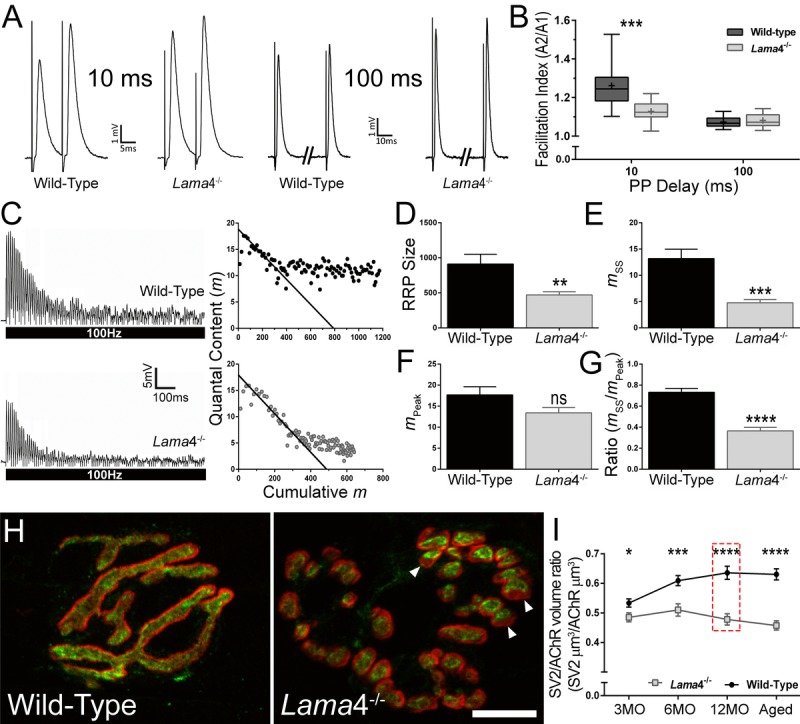
Lower facilitation and higher synaptic depression associated with decreased vesicle density at 12MO *lama*4^−/−^ neuromuscular junctions in EDL muscle (**A**) Sample traces of paired-pulse facilitation at 10 ms and 100 ms delay for 12MO WT and *lama*4^−/−^ NMJs. (**B**) Plot of facilitation ratio against paired-pulse delay times at 10 ms and 100 ms. (**C**) Representative EPPs recordings during 100 Hz stimulation for 1 s duration at 12MO WT and *lama*4^−/−^ NMJs. Estimation of RRP size was determined by plotting the y-axis (quantal content) over the x-axis (cumulative quantal content) and extrapolating the linear declining phase at x-axis. (**D**) RRP size, (**E**) quantal release during steady state plateau (*m*_ss_), (**F**) quantal release during the first ten stimuli (*m*_Peak_) and (**G**) ratio of *m*_ss_ to *m*_Peak_ measured between genotypes at 12MO. (**H**) Representative staining of synaptic vesicle 2 (SV2; green) with respect to postsynaptic AChRs endplate (red) at 12MO. *Lama*4^−/−^ NMJ clearly displayed lower degree of overlap between SV2 and postsynaptic AChRs endplate (as indicated by arrow heads) which was not observed in WT NMJ. Scale bar = 10 μm. **(I**) SV2/AChR volume ratio was measured across all ages investigated. The difference in SV2/AChR volume ratio between both genotypes was evident at 12MO (as indicated in red dashed line box). (**B**) *n* = 4, NMJs = 13-15 per genotype. (**D**-**G**), *n* = 4, NMJs = 12 for both genotypes. (**I**) *n* = 3, NMJs = 51-82 per genotype at each age group. Statistical analyses were performed using Student's *t*-tests for (**B**, **D**-**G**, and **I**); values are presented as mean ± SEM (* *P* < 0.05, ** *P* < 0.01, *** *P* < 0.001, and ****P < 0.0001).

Paired-pulse (PP) facilitation study was performed by comparing the second stimuli (test pulse) with the first stimuli (conditioning pulse) at 10 ms and 100 ms delays between each pulse, to determine the level of facilitation at 12MO NMJs. This age group was selected as *lama*4^−/−^ presented consistently lower levels of hind-limb grip strength. At 10 ms PP, WT NMJs demonstrated high facilitation with the amplitude of the second stimuli being 26.2% higher than the first response (Fig. 4A). On the other hand, *lama*4^−/−^ NMJs had a lower level of facilitation with the second response being 12.8% higher than the first (Fig. 4A). WTs produced significantly higher levels of facilitation compared with *lama*4^−/−^ NMJs (*P* = 0.0008; WT; 1.26 ± 0.03 *vs. lama*4^−/−^; 1.13 ± 0.02; Fig. 4B). The lower facilitation at *lama*4^−/−^ NMJs suggests an issue with the release of vesicles or a decrease in the availability of vesicles from the readily releasable pool (RRP). As the interval of PP was increased to 100 ms, the level of facilitation declined to a similar level in WT and *lama*4^−/−^ NMJs (Fig. 4A). No significant difference was found in facilitation between either genotype at 100 ms delay (*P* = 0.4837; WT; 1.07 ± 0.01 *vs. lama*4^−/−^; 1.08 ± 0.01; Fig. 4B). These findings suggest that under low demand (100 ms PP) *lama*4^−/−^ are capable of meeting signaling capacity, however perturbations appear when placed under high demand (10 ms PP), indicating a failure in transmission efficiency.

We next examined neurotransmission at 12MO *lama*4^−/−^ NMJs under high frequency stimulation (HFS). At 100 Hz stimulation for 1 s, WT displayed a gradual drop in EPP amplitude to reach a steady state plateau (Fig. 4C, top left). By contrast, *lama*4^−/−^ showed a rapid decline in EPP amplitude and reached a highly depressed steady state plateau (Fig. 4C, bottom left). We used a previously described model to estimate the size of RRP in both genotypes [23, 24]. Quantal content (*y*-axis) was plotted against cumulative quantal content (*x*-axis) and a straight line drawn in the declining phase to intercept the x-axis, providing an estimation of the RRP size (Fig. 4C, right panel). Quantal content was calculated for each EPP by correcting peak amplitude for non-linear summation and dividing it by the mean mEPP for the recording site [25]. *Lama*4^−/−^ NMJs (471.1 ± 45.0 quanta) had significantly smaller RRP size in comparison with WT (*P* = 0.0063; 912.3 ± 139.0 quanta; Fig. 4D). We then examined the peak quantal release, *m*_Peak_ which was measured during the first ten stimuli and found no significant difference (*P* = 0.0774) between WT (17.67 ± 1.93) and *lama*4^−/−^ NMJs (13.40 ± 1.26) suggesting that the delivery and release of vesicles during initial stimuli was normal in *lama*4^−/−^ (Fig. 4F). Analysis of quantal release during steady-state (*m*_ss_), found *lama*4^−/−^ NMJs were significantly lower than WT by 63.76% (*P* = 0.0002; WT; 13.19 ± 1.79 *vs*. *lama*4^−/−^; 4.78 ± 0.59), indicating either a lower availability of vesicles from the reserve pool or an issue with the delivery of vesicles from the reserve pool to RRP (Fig. 4E). Based on the ratio of *m*_ss_/*m*_Peak_, *lama*4^−/−^ were significantly lower than WT (*P* < 0.0001; WT; 0.73 ± 0.04 *vs. lama*4^−/−^; 0.36 ± 0.03), indicating impaired mobilization of vesicles during periods of sustained release (Fig. 4G).

We next investigated the expression of synaptic vesicle protein 2 (SV2) in order to examine the volume of synaptic vesicles at the presynaptic terminal in relation to the postsynaptic endplate. At 12MO, WT NMJs displayed densely packed vesicles which were colocalized to the postsynaptic endplates (Fig. 4H). By contrast, *lama*4^−/−^ NMJs showed an observable decrease in the overlap between SV2 staining in relation to the postsynaptic endplate (Fig. 4H), which matched the decline in transmission properties in both facilitation (Fig. 4A-B) and HFS studies (Fig. 4C-G). *Lama*4^−/−^ NMJs consistently had lower SV2/AChR volume ratios across all ages investigated when compared with WTs (3MO: *P* = 0.0216; WT; 0.53 ± 0.01 SV2 μm^3^/AChR μm^3^ *vs*. *lama*4^−/−^; 0.49 ± 0.02 SV2 μm^3^/AChR μm^3^, 6MO: *P* = 0.0003; WT; 0.61 ± 0.02 SV2 μm^3^/AChR μm^3^ *vs*. *lama*4^−/−^; 0.51 ± 0.02 SV2 μm^3^/AChR μm^3^, 12MO: *P <* 0.0001; WT; 0.64 ± 0.02 SV2 μm^3^/AChR μm^3^ *vs. lama*4^−/−^, 0.48 ± 0.02 SV2 μm^3^/AChR μm^3^ and Aged: *P <* 0.0001; WT; 0.63 ± 0.02 SV2 μm^3^/AChR μm^3^ *vs. lama*4^−/−^, 0.46 ± 0.02 SV2 μm^3^/AChR μm^3^; Fig. 4I). In contrast, WT NMJs gradually increased in SV2/AChR volume ratio with age and reached a plateau by 18-22 months (Fig. 4I).

### Accelerated aging of neuromuscular junctions in hind-limb muscles of *lama*4^−/−^ animals

Studies have found an association between changes in NMJ morphology as remodeling incidents or adaptation responses to altered neurotransmission [26, 27]. Our functional findings displayed impaired transmission properties, which may affect the general morphology of the NMJ at *lama*4^−/−^. In order to investigate this, we examined the innervation patterns of the nerve terminal in relation to the postsynaptic endplates in EDL muscles.

**Figure 5:**
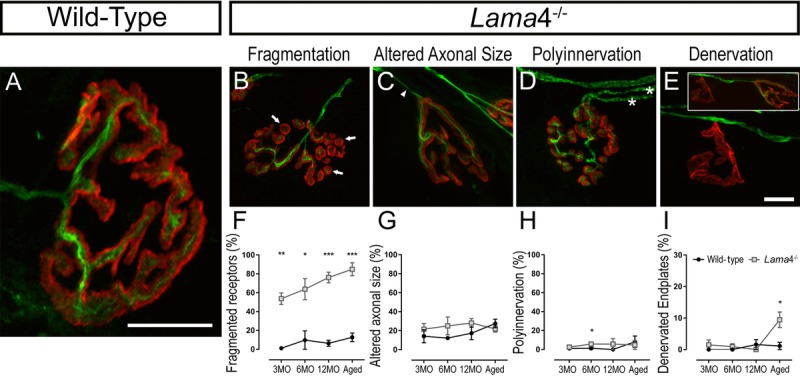
Accelerated aging features at *lama*4^−/−^ neuromuscular junctions in EDL muscle (**A**-**E**) Representative staining of axons and nerve terminal (green) in relation to the postsynaptic endplates (red) in WT (**A**) and *lama*4^−/−^ (**B**-**E**) at 3MO. WT (**A**) showed normal innervation pattern while *lama*4^−/−^ NMJs had (**B**) fragmented islands of postsynaptic receptors (as indicated by arrows), (**C**) altered innervating axonal size with thinning (as indicated by arrowhead) with some endplates displaying swollen axons, (**D**) polyinnervation or multiple axons entering the nerve terminal (as indicated with asterisks) and (**E**) completely denervated endplate with a smaller insert panel showing an occupied endplate alongside the denervated endplate. Scale bar = 10 μm. Quantification of aging features; (**F**) fragmented endplates, (**G**) altered axonal size, (**H**) polyinnervation and (**I**) denervated endplates across all ages. (**F**-**I**) *n* = 3, NMJs = 63-109 per genotype at each age group. Statistical analyses were performed using Student's *t*-tests; values are presented as mean ± SEM (* *P* < 0.05, ** *P* < 0.01, and ***P < 0.001).

Wild-type NMJs at 3MO showed mono-axonal innervation, with a single axon entering the nerve terminal which colocalized well with the pretzel shaped postsynaptic endplate (Fig. 5A). By contrast, *lama*4^−/−^ NMJs had higher proportion of fragmented postsynaptic endplate in comparison with WTs across the ages investigated (3MO: *P* = 0.0011; WT; 1.15 ± 1.15% *vs. lama*4^−/−^; 53.69 ± 6.11%, 6MO: *P* = 0.0220; WT; 9.80 ± 9.80% *vs. lama*4^−/−^; 63.78 ± 11.12%, 12MO: *P* = 0.0004; WT; 6.36 ± 3.19% *vs. lama*4^−/−^; 76.16 ± 5.63% and Aged: *P* = 0.0009; WT; 12.71 ± 4.55% *vs. lama*4^−/−^; 84.88 ± 6.84%; Fig. 5B and F). Endplates with altered innervating axonal size (i.e. axons prior to entry into the NMJ region), which takes into account of swollen and thinning axons, was similar at 3MO between both genotypes (*P* = 0.4574; WT; 14.12 ± 6.86% *vs. lama*4^−/−^; 21.60 ± 5.98%; Fig. 5C and G). By 6MO and 12MO, *lama*4^−/−^ innervating axonal size was consistently higher when compared with aged-matched WT axons although no significant difference was found (6MO: *P* = 0.2492; WT; 12.22 ± 1.71% *vs. lama*4^−/−^; 25.05 ± 9.37% and 12MO: *P* = 0.2522; WT; 17.34 ± 6.63% *vs. lama*4^−/−^; 28.12 ± 4.58%; Fig. 5C and G). Aged WT innervating axons (27.59 ± 4.48%) had a non-significant increase by 10.25% (*P* = 0.5563; one way ANOVA) when compared with 12MO WT axons, and had reached the similar level of altered axonal size with aged *lama*4^−/−^ NMJs (*P* = 0.3668; 21.91 ± 3.33%; Fig. 5G).

*Lama*4^−/−^ NMJs presented similar proportion of polyinnervated endplates with WT NMJs at 3MO (*P* = 0.4704; WT; 1.15 ± 1.15% *vs. lama*4^−/−^; 2.56 ± 1.34%; Fig. 5D and H). At 6MO and 12MO, *lama*4^−/−^ NMJs had higher number of polyinnervated endplates in comparison with aged-matched WT NMJs (6MO: *P* = 0.0452; WT; 1.28 ± 1.28% *vs. lama*4^−/−^; 5.77 ± 0.89% and 12MO: *P* = 0.3739; WT; 0% (no polyinnervated endplates observed) *vs. lama*4^−/−^; 5.71 ± 5.71%; Fig. 5D and H). When aged, proportion of polyinnervated endplates in WTs had increased to the similar level of *lama*4^−/−^ NMJs (*P* = 0.6863; WT; 8.12 ± 6.07% *vs. lama*4^−/−^; 4.76 ± 4.76%; Fig. 5D and H). Interestingly, *lama*4^−/−^ NMJs showed a different trend in denervated endplates from the other features discussed. Denervated endplates remained low in proportion at both genotypes from 3MO to 12MO, however at aged NMJs there was a significant increase in the number of denervated endplates at *lama*4^−/−^ NMJs when compared with age-matched WT NMJs (*P* = 0.0371; WT; 1.15 ± 1.15% *vs. lama*4^−/−^; 9.48 ± 2.45%; Fig. 5E and I).

### Altered postsynaptic endplate morphology at *lama*4^−/−^ neuromuscular junctions

The high prevalence of fragmented receptors warranted further investigation of the postsynaptic AChR endplates. We measured the postsynaptic endplates using different parameters such as stained endplate area only, expanded region of the endplate and dispersion of AChRs, as these parameters may provide greater details on the extent of the remodeling incidents in these mice [14, 28, 29].

**Figure 6:**
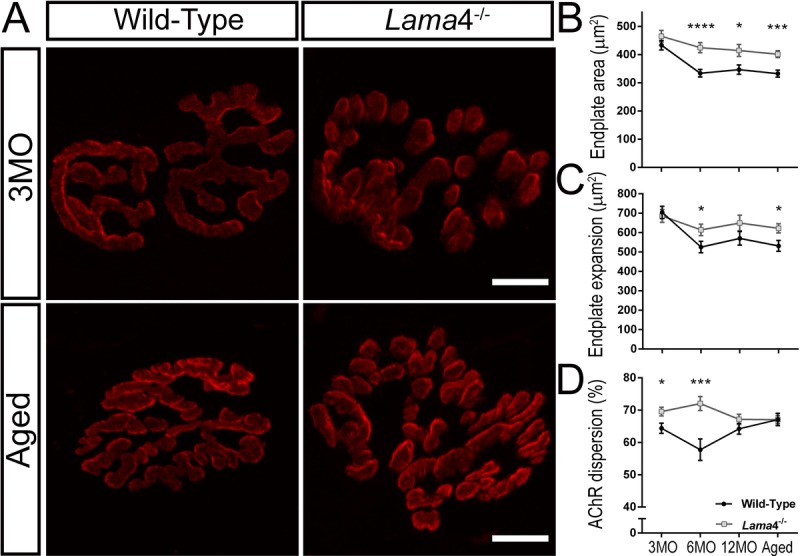
Larger postsynaptic endplates in area, expansion and dispersion of AChRs at *lama*4^−/−^ neuromuscular junctions (**A**) Representative staining of postsynaptic AChR endplates (red) in EDL muscle. WT and *lama*4^−/−^ NMJs at 3MO had similar synapse area and endplate expansion. Aged *lama*4^−/−^ NMJs maintained large synapse area and expansion similarly to 3MO whereas aged WT appeared smaller in area and expansion in comparison with both genotypes at 3MO and aged *lama*4^−/−^ NMJs. Scale bar = 10 μm. Quantification of (**B**) synapse area, (**C**) endplate expansion and (**D**) dispersion of AChRs across all age groups. (**B**-**D**) *n* = 3, NMJs = 51-82 per genotype at each age group. Statistical analyses were performed using Student's *t*-tests; values are presented as mean ± SEM (* *P* < 0.05, *** *P* < 0.001, and ****P < 0.0001).

At 3MO, WT and *lama*4^−/−^ NMJs had similar synapse area (*P* = 0.2390, WT; 433.1 ± 16.8 μm^2^ *vs. lama*4^−/−^; 464.9 ± 20.7 μm^2^) and expanded region of the postsynaptic endplate (*P* = 0.6903, WT; 704.0 ± 31.5 μm^2^ *vs. lama*4^−/−^; 685.8 ± 33.1 μm^2^; Fig. 6A-C). Interestingly, *lama*4^−/−^ NMJs (69.55 ± 1.33%) at this age displayed significantly larger dispersion of AChRs in comparison with WTs (*P* = 0.0132; 64.39 ± 1.58%; Fig. 6D). By 6MO, WTs declined significantly in both synapse area (*P* < 0.0001, 333.7 ± 13.3 μm^2^; one way ANOVA) and endplate expansion (*P* = 0.0002, 525.8 ± 29.5 μm^2^; one way ANOVA) from 3MO WTs, and also significantly smaller in both of these parameters when compared with 6MO *lama*4^−/−^ (synapse area; *P* < 0.0001, 424.8 ± 17.87 μm^2^ and endplate expansion; *P* = 0.0372, 614.0 ± 29.78 μm^2^; Fig. 6B and C). *Lama*4^−/−^ NMJs (72.06 ± 2.16%) at this age remained significantly larger in AChRs dispersion in comparison with WT (*P* = 0.0004; 57.76 ± 3.32%; Fig. 6D).

Both genotypes maintained similar trends at 12MO and aged groups with *lama*4^−/−^ NMJs presenting larger synapse area (12MO: *P* = 0.0139; WT; 346.7 ± 16.4 μm^2^ *vs. lama*4^−/−^; 414.5 ± 21.2 μm^2^, Aged: *P* = 0.0001; WT; 332.5 ± 12.35 μm^2^ *vs. lama*4^−/−^; 401.5 ± 11.98 μm^2^) and larger expanded endplate region (12MO: *P* = 0.1420; WT; 570.5 ± 35.0 μm^2^ *vs. lama*4^−/−^; 649.9 ± 40.1 μm^2^, Aged: *P* = 0.0148; WT; 531.8 ± 27.86 μm^2^ *vs. lama*4^−/−^; 622.0 ± 23.93 μm^2^; Fig. 6A-C). Interestingly, WT at 12MO (64.23 ± 1.65%) increased in dispersion of AChRs almost to the similar level of *lama*4^−/−^ NMJs (*P* = 0.2007; 67.16 ± 1.58%; Fig. 6D). At aged group, WT endplates (67.10 ± 1.89%) had reached the similar level of AChRs dispersion as *lama*4^−/−^ NMJs (*P* = 0.9760; 67.03 ± 1.24%; Fig. 6D).

### Altered expression of laminin-α4 at wild-type neuromuscular junctions during aging

**Figure 7:**
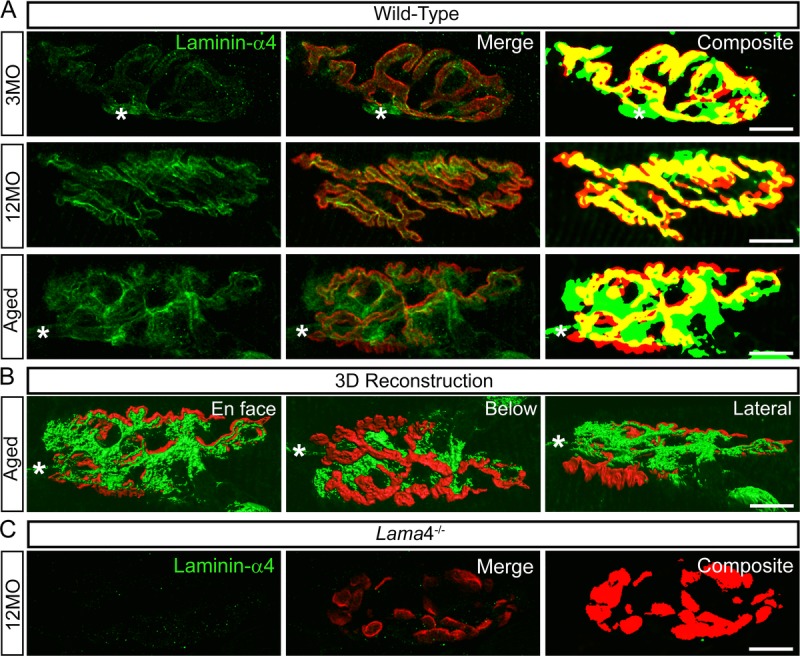
Altered expression of laminin-α4 at 12MO wild-type NMJs, becoming more evident at aged neuromuscular junctions (**A**) Examples of representative staining of laminin-α4 chain (green) with respect to the postsynaptic endplates (red) in EDL muscle. Composite panel showed colocalization of laminin-α4 with postsynaptic endplate = yellow, extrasynaptic localization of laminin-α4 = green and missing laminin-α4 expression at postsynaptic region = red. Asterisk (*) is an indication of a nerve entering the nerve terminal, therefore it is not considered as an extrasynaptic localization of the laminin-α4 chain. WT at 3MO showed normal colocalization of laminin-α4 chain with respect to the postsynaptic endplates. By 12MO, WT began to display missing expression of laminin-α4 (observed with more red on composite panel) and mislocalized laminin-α4 expression (more green) compared with 3MO WT. As the NMJ aged, WT showed drastic changes in laminin-α4 expression with highly expressed laminin-α4 at the extrasynaptic region (high degree of green) and lacking laminin-α4 (high degree of red) compared with 12MO WT. The overlapping of laminin-α4 to postsynaptic endplates (yellow) at aged WT NMJ was at a much lesser degree in comparison with 12MO WT. (**B**) 3-dimensional (3D) reconstruction of laminin-α4 staining (green) in relation to postsynaptic endplate (red) of aged WT NMJ from (**A**), in three different planes of view; *en face*, below and lateral. *En face* view is reflective of the image shown in (**A**). Below and lateral views confirmed that non-colocalized laminin-α4 staining was purely expressed at the extrasynaptic basal lamina region, not by non-muscle cells. (**C**) *Lama*4^−/−^ NMJs at 12MO displayed no expression of laminin-α4 chain. Scale bar = 10 μm.

Aged WT NMJs displayed functional, behavioral and morphological characteristics similar to those observed at *lama*4^−/−^ NMJs. This suggests that these alterations at WT NMJs may be associated with a change in expression of laminin-α4 leading to a loss in NMJ integrity and efficiency. We therefore examined laminin-α4 staining at NMJs from WT mice, with the omission of 6MO as this group consistently presented functional and morphological characteristics similar to the 3MO age group. At 3MO, WT NMJs displayed normal expression and colocalization of laminin-α4 with respect to the postsynaptic endplates (Fig. 7A), which coincided with normal neurotransmission and morphology seen in WT NMJs at this age group. By 12MO, WT NMJs displayed mislocalized laminin-α4 expression extrasynaptically as well as postsynaptic endplate regions void of laminin-α4 expression (Fig. 7A). These alterations became further evident in aged WT NMJs (Fig. 7A and B), and coincided with the prominent changes observed functionally, behaviorally and morphologically at this age.

## DISCUSSION

Previous studies have demonstrated the premature appearance of morphological features associated with aging at 6MO *lama*4^−/−^ NMJs. The early appearances of these features implicate laminin-α4 in maintaining a normal healthy NMJ during adulthood and aging. The present study further explored this theory by examining the role of laminin-α4 in maintenance using functional transmission, morphological investigation of synaptic molecules and behavioral characterization. Our findings demonstrated non-progressive defects in transmission at *lama*4^−/−^ NMJs consistent with the changes seen in synaptic proteins. We also observed non-progressive weaker hind-limb grip force in *lama*4^−/−^ animals. Interestingly, aged (18-22MO) WTs declined in both transmission and behavioral movement to levels comparable to *lama*4^−/−^. Furthermore, missing and mislocalized expression of laminin-α4 were noted at WT NMJs before the evident declines in transmission and behavioral movement occurred. These findings strongly support the role of laminin-α4 in maintenance of the NMJ during aging.

### Non-progressive defects in neurotransmission and morphology despite accelerated aging neuro-muscular junctions in *lama*4^−/−^

Morphological studies demonstrated early aging features at 6MO *lama*4^−/−^ NMJs which increased with age in diaphragm muscles [9]. Interestingly, our functional findings in this muscle demonstrated non-progressive disrupted transmission properties despite the worsening of aging features observed by others in these mutants. Our observations demonstrated consistently lower frequency in spontaneous release and higher failures in evoked release across each age group investigated at *lama*4^−/−^ NMJs. Furthermore, these mutant NMJs showed decreased quantal content which was due to the lower number of active release sites, suggesting alterations at the presynaptic nerve terminal as the contributor to impaired neurotransmission. This is consistent with our morphological findings which showed the decline in the density of the active zone marker Bassoon at *lama*4^−/−^ NMJs, which remained at similar levels from 3MO to aged mice. Interestingly, a study showed no change in active zone number at adult *lama*4^−/−^ NMJs [12]. This discrepancy in results could be explained by the different techniques used to quantify the active zone number. The study utilized electron microscopy which only took into account of a single section of the synapse and therefore may not provide a true representation of the entire structure as only a small number of active zones would be accounted for quantification [12]. In the present study, we employed wholemount immunostaining, which allowed for staining of active zones over the entire endplate region providing a more accurate reflection of active zone distribution across an entire NMJ.

The increased failures in evoked release at mutant NMJs may have been due to the intermittent failure in action potential propagation to the nerve terminals, as studies have found altered myelination patterns at the perineural sheathing of the peripheral nerve when laminin-α4 is absent at this region [6, 30]. However, our findings demonstrated consistent presence of the NTI at *lama*4^−/−^ NMJs even though failures in evoked release had increased, suggesting normal propagation of action potentials to the nerve terminal. Therefore, a higher failure in evoked release was directly as a result of defects at the level of depolarization-secretion coupling at *lama*4^−/−^ NMJs. What is interesting here is the observation that transmission and morphological changes in *lama*4^−/−^ NMJs although initially perturbed at 3MO, do not show an age-dependent improvement or decline [9]. The maintained level of transmission may be due to counter adaptive mechanisms inherent at the highly plastic NMJ.

### Weaker grip force in *lama*4^−/−^ was not due to changes in fiber type composition

We observed functional and morphological changes that remained constant from 3MO to aged *lama*4^−/−^ NMJs. Here, we also observed similar trends in hind-limb grip force, with *lama*4^−/−^ consistently producing weaker force in comparison with WTs. In support of our findings of altered neuromuscular function, a previous study observed uncoordinated hind-limb movement in *lama*4^−/−^ mice [12]. The consistently weakened grip force suggests a non-progressive defect, which is in agreement with the coordination and body balance studies previously conducted [12]. Weaker grip force could result from alterations in fiber type compositions involving increased proportion of slow fiber types (Type I) with downregulation of fast fiber types [31, 32]. In order to determine this, we looked at fiber type expression in gastrocnemius muscles at 3MO and aged mice. *Lama*4^−/−^ displayed normal fiber type composition at both age groups suggesting weaker grip force in these animals is not due to altered fiber type expression, but rather a result of impaired neurotransmission.

### Disrupted transmission in hind-limb muscles is associated with decreased vesicle availability at *lama*4^−/−^ neuromuscular junctions

As we observed weaker hind-limb grip force in *lama*4^−/−^, we examined the capacity of NMJs to sustain transmitter release during paired-pulse facilitation and higher frequency stimulation of the hind-limb EDL muscle at 12MO. *Lama*4^−/−^ displayed lower facilitation which may reflect an issue with the release of vesicles from the RRP. Our findings on younger P18 *lama*4^−/−^ NMJs displayed normal presence of vesicular associated proteins, such as synapsin 1, syntaxin 1A and SNAP25 [33]. This suggests that while these proteins may be present, the interaction between them may be dysfunctional and thus interfering with the proper release of vesicles during facilitation. Alternatively, perturbed neurotransmission may have resulted from a decrease in the availability of synaptic vesicles, which is consistent with our morphological findings that showed lower SV2/AChR volume ratios at *lama*4^−/−^ NMJs. In addition, it is also possible that lower facilitation occurred as a result of higher release probability. However, based on our binomial analysis, no significant changes were observed in release probability at *lama*4^−/−^ NMJs.

Transmitter release from NMJs of both genotypes underwent depression and reached a steady-state plateau after high frequency stimulation, however *lama*4^−/−^ NMJs displayed a rapid decline and lower plateau in comparison with WT NMJs. This rapid depression may be due to a decrease in the number of active release sites (*n*) and therefore reduced vesicle availability or an compensatory increase in the probability of release (*p*) in *lama*4^−/−^. Our binomial analysis showed no significant changes in *p*, however we did observe a drop in *n*. The rapid drop in transmitter release suggests a faster depletion of vesicles from the RRP, which matched the smaller estimated size of RRP at *lama4*^−/−^ NMJs [23, 24, 34-36]. The lower plateau of neurotransmitter release reflects issue with the replenishment of synaptic vesicles from the reserve pool which could be explained by the lower number of vesicles available from this pool [23, 24, 34-36]. Indeed, our morphological findings support this idea and are consistent with the observed functional changes during conditions of high demand in transmitter release.

### Remodeling occurrences associated with morphological changes at *lama*4^−/−^ neuromuscular junctions

At 3MO *lama*4^−/−^ NMJs, we observed a high incidence of fragmented AChRs in EDL muscles which is a characteristic feature of NMJ degeneration and premature aging. Previous study has shown this feature became prominent by 6MO in the diaphragm muscle of *lama*4^−/−^ mice [9]. The discrepancy between these findings may partly be attributed to the fact that the EDL is comprised primarily of fast fiber types, and is therefore more susceptible to degeneration or remodeling of the NMJ [13], while the diaphragm is a mixed fiber type muscle and is more resistant to changes at the NMJ [26]. We also observed a significantly higher proportion of fully denervated NMJs at aged *lama*4^−/−^ EDL muscles. Therefore, it is likely that loss in laminin-α4 results in accelerated remodeling of the NMJs in EDL muscles. We propose that loss of laminin-α4 results in constant NMJ remodeling in the EDL muscle, which is initially maintained at a sufficient level to keep the NMJ active and intact. However, due to the high energy demand of these remodeling processes it cannot be maintained, resulting in full retraction of the nerve terminal from the endplate and eventually degeneration of the NMJ.

Alterations in the morphology of the postsynaptic AChR endplate were also observed, with increased AChR area, expansion and dispersion at *lama*4^−/−^ NMJs. Alterations to endplate morphology may be associated with compensation of a failing NMJ and/or potentially remodeling of the synapse [14, 28, 29]. It is plausible that failing transmission at *lama*4^−/−^ NMJs results in counter-adaptive mechanisms employing the addition of AChRs to the endplate region, providing more receptors for released transmitter to bind to and consequently increase the likelihood of signal propagation, which may partly explain the higher amplitudes seen in these mice. Studies have found that neuromuscular inactivity tested through the blockade of synaptic transmission by tetradotoxin leads to larger endplate areas [37], while blockade of presynaptic transmission by Botulinum toxin resulted in greater dispersion of the existing AChRs as well as addition of newly formed AChRs at the postjunctional folds [28]. Similar occurrences were also observed when AChRs were subjected to blocking with bungarotoxin or curare [38]. We propose that the higher failures of transmitter release and decrease in spontaneous frequency at *lama*4^−/−^ NMJs mimics that of neuromuscular inactivity, resulting in synaptic remodeling of the postsynaptic AChR endplate as a compensatory mechanism to enhance neurotrans-mission.

### Laminin-α4 is required for the maintenance of the adult neuromuscular junction

Our findings indicate an association between the loss of laminin-α4 at the NMJ and perturbed transmission, morphology and behavioral movement from adulthood to aging. Here we asked whether alterations in laminin-α4 expression at WT NMJs may be related to the functional neurotransmission and behavioral changes noted in aged mice. We observed mislocalized and absent laminin-α4 expression at 12MO WT NMJs which preceded any prominent signs of decline in neurotransmission. At aged WT NMJs, the changes in laminin-α4 expression became more evident and coincided with decreased transmission properties and weakened hind-limb grip force to levels resembling that of *lama*4^−/−^ NMJs. Based on these observations, it appears that changes in laminin-α4 expression at WT NMJs preceded the alterations in its functional transmission, and these alterations resembled the trends evident at *lama*4^−/−^ NMJs. These findings support the idea that laminin-α4 is required for the maintenance of the adult NMJs.

At aged WT NMJs, we also noted a significant decrease in the density of the active zone marker Bassoon, consistent with prior study investigating active zones at aged NMJs [39, 40]. This decrease in active zones coincided with the altered laminin-α4 expression, suggesting a role for laminin-α4 in aligning presynaptic apparatus to postjunctional folds, and thus aiding the NMJ maintenance. Interestingly, we observed an increase in SV2/AChR volume ratios at aged WT NMJs, which were in contrast to subtly decreased volume ratios at *lama*4^−/−^ nerve terminals. Our findings are consistent with others that show a relative increase in synaptic vesicles at aged terminals [41, 42]. We propose that the lower SV2/AChR volume ratios in *lama*4^−/−^ nerve terminals is a maintained phenotype from early perturbations of neurotransmission, while the increase in volume ratio at WT NMJs is a result of adaptive synaptic remodeling. The increased SV2/AChR volume ratio may be a remodeling mechanism as a result of inefficient transmitter release at a failing terminal. Prior studies have shown that disuse of muscles through hind-limb suspension in aged animals resulted in the expansion of synaptic vesicles as an adaption or remodeling process at the synapses [43]. Furthermore, another study demonstrated accumulation of vesicles resulted from failure in synaptic transmission [44], which supports our findings as our aged control NMJs had increased failures in evoked release. We propose that altering the amount of vesicles as an adaptive response is more feasible than altering the number of active zones in a neuromuscular system when transmission properties decline in aging WT NMJs.

## CONCLUSION

Loss of laminin-α4 leads to non-progressive impaired neurotransmission and premature morphological alterations normally associated with an aging NMJ. Aged WT NMJs demonstrated similar functional and morphological characteristics to those observed at *lama*4^−/−^ NMJs. Most importantly, alterations in laminin-α4 expression at WT NMJs occurred prior to the appearance of these functional impairments, suggesting that changes in laminin-α4 expression precede changes associated with aging and may therefore be a critical mediator in determining the survivability of the NMJ. Our results show the significant role of laminin-α4 in maintaining functional capacity and morphological organization of the adult NMJ during aging. These findings indicate that laminin-α4 has an important role in not only maintaining neuromuscular structure and function but possibly also the maintenance of muscle function during aging. Maintenance of healthy adult NMJs may delay onset of aging phenotype in skeletal muscle that would help improve the health of the elderly. Laminin-α4 may act as a biomarker of NMJ integrity during aging and disease states, and might therefore serve as a potential target for therapeutic treatment to delay the onset of functional decline observed under these conditions.

## MATERIALS AND METHODS

### Ethics

The University of Queensland Animal Care and Ethics Committee approved all procedures undertaken (Ethics number 188/15) and were in accordance with the Queensland Government Animal Research Act 2000, associated Animal Care and Protection Regulations (2002 and 2008), as well as the Australian Code for the Care and Use of Animals for Scientific Purposes, 8^th^ Edition (National Health and Medical Research Council, 2013).

### Animals

Wild-type mice (with two normal copies of all laminin genes) and homozygous mutant mice (with no normal copies of the *lama*4 gene) were used in this study. Knockout mice were obtained from the mating of homozygous mutant males and females and maintained on a defined C57BL/6-129SvJ genetic background. Animals were genotyped using a tail tip DNA tail assay [45]. Mice were used at the age groups of 3 months old (3MO), 6 months old (6MO), 12 months old (12MO) and 18-22 months old (Aged). Mice were anaesthetized with rising concentrations of carbon dioxide and then euthanized by cervical dislocation. A minimum of three animals was used at each age group for electro-physiology, histological procedures and behavioral studies detailed below.

### Electrophysiology

#### Tissue preparation

Mouse diaphragm muscle with intact phrenic nerve was dissected free and pinned to the bottom of a Sylgard-coated recording chamber. The preparation was perfused at a rate of 3 mL min^-1^ with Tyrode's solution (composition (mM): NaCl 123.4; KCl 4.7; NaH_2_PO_4_ 1.3; NaHCO_3_ 16.3; MgCl_2_ 1.0; glucose 7.8; CaCl_2_ 0.3-2.0; pH 7.3). The reservoir supplying the bath was continuously gassed with 95% O_2_ and 5% CO_2_ and maintained at room temperature 22 ± 2^0^C. EDL muscles with intact peroneal nerves were dissected and prepared similarly as the diaphragm muscles.

#### Electrical stimulation

The phrenic nerve supplying one hemi-diaphragm was sucked into a glass pipette filled with Tyrode's solution. This pipette acted as a stimulating electrode utilizing two silver chloride wires, one passing within the pipette as a positive electrode and the other outside as the negative electrode. The phrenic nerve was stimulated with square wave pulses of 10-20 V intensity, 0.08 ms duration at a frequency of 0.2 Hz, using a Grass Instruments stimulator (SD48) coupled to a Grass stimulus isolator (SIU5). This was performed similarly on EDL muscles, but stimulated at a frequency of 0.5 Hz with 0.1 ms duration at 10 or 100 ms delay for short term facilitation studies. For high frequency studies the EDL was stimulated using 100 Hz trains of 1 s duration.

#### Extracellular recordings

Extracellular recordings of nerve terminal impulses (NTIs), endplate currents (EPCs) and miniature endplate currents (mEPCs) were obtained, as previously described [46]. In brief, coarse glass micropipettes (2-10 μm in diameter) filled with Tyrode's solution were positioned on the surface of muscle fibers until sharp rise times of less than 1 ms were detected for both EPCs and mEPCs. The frequency of EPCs and mEPCs were carefully monitored while the electrode was lowered as electrode pressure may give rise to increased frequency of spontaneous activity [47-49]. After a recording site was located, the stimulus was ceased for a five-minute period before commencing recordings of EPCs and mEPCs. This allowed for replenishment of vesicular pools at the nerve terminals. Recorded signals were amplified using an Axoclamp 2B amplifier (Axon Instruments, USA) and digitized to 20-40 kHz sampling rate using MacLab system and Scope software (Version 3.5.5, AD Instruments, CO, USA). Five to six sites were selected from each muscle preparation with recording between 10-15 minutes. Extracellular recordings were used to determine amplitudes of both EPCs and mEPCs as well as NTIs.

#### Intracellular recordings

Recordings were performed as per our previous study [46]. In brief, fine tipped glass microelectrodes (30 to 50 MΩ) filled with 2 M KCl were used to record endplate potentials (EPPs), miniature endplate potentials (mEPPs) and resting membrane potentials (RMPs). Electrodes were manipulated to impale muscle fibers within 0.5 mm of the endplate region. Proximity to endplate regions was confirmed through analysis of EPP and mEPPs rise times, sites with rise times greater than 1.5 ms were rejected. During recordings the initial RMP values were in the range of 70-85 mV with these values undergoing a gradual decrease to steady values between 55-60 mV. Recordings were terminated if the RMP fluctuated by more than 10% from the steady values. After a site was located stimulus was ceased for a rest period of ten minutes. The sodium channel blocker, μ-Conotoxin GIIIB (0.5-2 μM, Alomone Labs) was used to prevent stimulus-induced muscle contractions at elevated [Ca^2+^]_O_.

#### Paired pulse facilitation

The facilitation of WT and mutant NMJs was investigated by applying twin pulses at 10 and 100 ms delays with Tyrode's solution containing 2 mM [Ca^2+^] and 1 mM [Mg^2+^]. The degree of facilitation was evaluated using the facilitation index (*fi*): *fi* = A_2_/A_1_ where A_2_ is the average of the EPP amplitudes elicited by the second stimulus and A_1_ is the average of the EPP amplitudes elicited by the first stimulus [50]. A total of 4 WT mice (NMJs = 15) and 4 *lama*4^−/−^ mice (NMJs = 13) were utilized for this study.

### Wholemount immunohistochemistry

Hemi-diaphragm and EDL muscles were used from WT and mutant mice at all age groups investigated. The muscles were incubated with Alexa Fluor 555-alpha bungarotoxin (α-BTX; Molecular Probes, Invitrogen, Eugene OR, USA) diluted at 1:1000 in phosphate buffered saline (PBS) followed by fixation with 2% paraformaldehyde (PFA)/PBS and then washed with PBS. The tissues were blocked with either 2% bovine serum albumin (BSA), 2% goat serum and 0.5% Triton X-100 (TX-100)/PBS or 2% BSA and 0.5% TX-100/PBS followed by washing with 0.5% TX-100. The tissues were then incubated with the following primary antibodies overnight at 4^0^C; mouse anti-Bassoon (1:300 dilution; Enzo Life Sciences; SAP7F407 clone; [7, 46, 51]), mouse anti-SV2 developed by Buckley K.M. was obtained from the Developmental Studies Hybridoma Bank (DSHB), created by the NICHD of the NIH and maintained at The University of Iowa, Department of Biology, Iowa City, IA 52242 (1:200 dilution; [52]) and combination of mouse anti-SV2 (1:200 dilution) plus rabbit anti-neurofilament (1:500 dilution: Sigma Aldrich; N41402; [53]). The tissues were washed with 0.5% TX-100 prior to incubation with appropriate Alexa Fluor 488-conjugated secondary antibodies (1:1000 dilution; Molecular Probes, Invitrogen) for 5 hours at 4^0^C. Muscles were then washed with PBS and mounted in Prolong Gold antifade reagent (Molecular Probes, Invitrogen) and cover-slipped. All antibodies were diluted in 0.5% TX-100.

### Cryosection immunofluorescence for the detection of laminin-α4 chain

Muscles were fixed with 4%PFA/PBS, washed with PBS and then immersed consecutively in 15% sucrose/PBS overnight followed by 30% sucrose/PBS overnight. The muscles were embedded in blocks with optimal cutting temperature (OCT) medium and snapped frozen in liquid nitrogen. The frozen blocks were longitudinally sectioned at 30 μm with a cryostat. Cryosections were blocked with 2% BSA and 0.1% TX-100/PBS followed by incubation with rabbit anti-laminin-α4 (ID# 1100; 1:500 dilution) at 4^0^C overnight (antibody kindly provided by Professor Jeffrey Miner). After primary antibody incubation, tissues were incubated in combination of Alexa Fluor 555 α-BTX (1:5000) with Alexa Fluor 488 goat anti-rabbit (1:1000 dilution) for 2 hours at room temperature. Sections were later washed with PBS consecutively and mounted with Prolong Gold antifade reagent. All antibodies and α-BTX were diluted in 2% BSA and 0.1% TX-100/PBS.

### Fibre typing

Gastrocnemius muscles were embedded in OCT medium and freshly snapped frozen. Frozen blocks of muscles were cut transversely at 10 μm in the mid-belly region using cryostat. Fibre type staining was performed as previously described [54]. The cryosections were firstly blocked with mouse on mouse (M.O.M.) IgG blocking buffer (Vector Lab)/PBS for 1 hour before incubated with the following primary antibodies overnight at 4^0^C; mouse anti-BA-D5 which stains the myosin heavy chain Type I at 1:100, mouse anti-SC-71 which stains the myosin heavy chain Type IIA at 1:100, and mouse anti-BF-F3 which stains the myosin heavy chain Type IIB at 1:80. These primary antibodies were deposited to the DSHB by Schiaffino, S and diluted in 0.5% BSA/PBS. The remaining unstained fibres were grouped as Type IIX, as noted by other researchers [54, 55]. The tissues were then incubated for 1 hour at room temperature with the appropriate specific secondary antibodies in combination of Alexa Fluor 647 AffiniPure Goat Anti-mouse IgG, Fcy subclass 2b specific, Alexa Fluor 594 AffiniPure Fab Fragment Goat Anti-mouse IgM, u chain specific and Alexa Fluor 488 AffiniPure Goat Anti-mouse IgG, Fcy subclass 1 specific (1:200 dilution). All secondary antibodies were purchased from Jackson Immunoresearch, and diluted in 0.5% BSA and 2% goat serum/PBS. Cryosections were then washed with PBS, mounted with Prolong Gold antifade reagent and cover-slipped.

### Image acquisition and analysis

Tissues were imaged with an Olympus Fluoview FV1000 confocal laser scanning microscope, equipped with two excitation diode laser lines (473 nm and 559 nm) running on Fluoview FV10-ASW software version 01.07C. Images were taken at a resolution of 1024 by 1024 pixels in *xy*, using a 100x/ 1.35 NA Oil Iris UPlan-Apochromat objective with a Z-step size of 0.3 μm, providing a voxel size of 0.124 x 0.124 x 0.3 μm^3^. Images were captured using identical laser power levels, photomultiplier gain levels, scanning speed and pinhole size amongst different slides using the same antibody. Imaris 8.1.2. (Bitplane, South Windsor, CT, USA) was used to analyze Z-stacked images for volume measure-ment of synaptic vesicles and postsynaptic endplate as previously conducted [33, 56]. Synaptic vesicles targeted by mouse anti-SV2 stained with Alexa Fluor 488-conjugated secondary antibody and postsynaptic AChR endplates stained with Alexa Fluor 555 α-BTX, were rendered in 3D based on fluorescent intensity using the surface-rendering module as previously described [33, 56, 57]. The mouse anti-SV2 antibody is highly specific for the binding of synaptic vesicles and it has been widely used for the staining of synaptic vesicles in other studies [33, 52, 56]. Interactive histogram based volumetric pixels was used for thresholding of both SV2 and AChR labelling in order to accurately render 3D solid reconstructions without creating artifacts [33, 57]. Imaris software then performed automated volume calculation based on rendered 3D structures for both SV2 and postsynaptic AChR endplates. The SV2/AChR volume ratio at each junction was determined by dividing the volume of the 3D reconstructed SV2-stained regions by the 3D reconstructed endplate volumes. The endplate volume is defined as the entire 3D reconstructed endplate inclusive of interstitial spaces. Postsynaptic AChR endplates from these captured images were also measured for a number of different parameters such as synapse area (stained AChRs only), endplate expansion area (the surrounding exterior region of the stained postsynaptic endplate) and dispersion of AChRs (measured through the division of synapse area over endplate expansion area followed by multiplication with 100 [58]). Tissues labelled for the detection of nerve terminal and axons with respect to the postsynaptic endplates were imaged with the same microscope above. Selected fields were imaged with a 40x/0.95 NA Uplan-Apochromat objective with a Z-step size of 0.5 μm, providing a voxel size of 0.31 x 0.31 x 0.5 μm^3^. The criteria for scoring of aging features was performed as previously described [59], with 1) fragmented receptors containing 5 or more AChR islands and/or a region of the postsynaptic AChR displays significant morphological abnormalities such as irregularly shaped AChR clusters, 2) axonal swelling as bulging or enlargement of the axon proximal to the postsynaptic AChRs, 3) polyinnervation with two or more axons entering a single postsynaptic AChR and 4) denervation with postsynaptic endplate completely unapposed by nerve terminal. Based on our criteria, axonal thinning was scored when innervating motor axon is less than 1 μm in diameter. Tissues stained for fibre types were also imaged with Olympus Fluoview FV1000 confocal microscope, using a 10x/0.40 NA UPlan-Apochromat objective.

Composite images for laminin-α4 staining were generated using a modified protocol to that previously described [60]. In brief, image Z-stacks were projected into single maximum intensity projections using Image J software [61]. Background was subtracted with the rolling ball radius adjusted to appropriate pixel values so as to not to interfere with true signal (50-80 pixels). Following this process, images underwent noise despeckling and a standard Gaussian Blur filter (σ = 2.00) was applied. A colour threshold was then applied to the presynaptic laminin stained region and next to the postsynaptic AChR stained region with a Moments thresholding method. Thresholding was adjusted to ensure the relevant regions were superimposed by the selected thresholding method. Composite overlays were created by merging individual channels. This process was conducted on images that best represent the morphological phenotypes qualitatively described in text. In addition to this, 3D reconstruction of laminin-α4 staining with respect to postsynaptic endplate was conducted using Imaris software, on aged WT NMJ as similarly performed for 3D reconstruction of synaptic vesicles and postsynaptic endplates.

Tissues labeled by presynaptic active zone marker, Bassoon were imaged with a spinning-disk confocal system (Marianas; 3I, Inc.) consisting of a Axio Observer Z1 (Carl Zeiss) equipped with a CSU-W1 spinning-disk head (Yokogawa Corporation of America) and ORCA-Flash4.0 v2 sCMOS camera (Hamamatsu Photonics). Image acquisition was performed using SlideBook 6.0 (3I, Inc) with a Z-step size of 0.13 μm, using 100x 1.4 NA PlanApo objective, providing a voxel size of 0.063 x 0.063 x 0.13 μm^3^. The captured images were deconvolved with Huygens Professional version 16.05 (Scientific Volume Imaging, The Netherlands), using the CMLE algorithm, to improve resolution and signal to noise ratio for improved analysis [62]. Imaris 8.1.2 was used to analyze the images for counts of Bassoon puncta and volume of postsynaptic endplate [46, 57]. The degree of sensitivity in detecting Bassoon puncta was adjusted using an automatically-generated interactive histogram based on voxel size and the minima determined by the nominated puncta diameter of 0.2 μm [40, 57]. The density of Bassoon for each junction was then determined by dividing the total number of Bassoon puncta over the reconstructed endplate volumes. All image analysis was completed under blinded conditions.

### Hind-limb grip force test

Before commencing the test of hind-limb grip force, mice were weighed for their body weight. A DPS-0.5R digital force gauge (Imada, Japan) with extension shaft and T-bar was used to measure maximal hind-limb grip force. Mice were held by their tail and lowered until their hind-limbs grip the T-bar that is connected to the digital force gauge. Once the mice were positioned horizontally, they were pulled away from the bar with consistent pull in order to release their grips off the T-bar. The hind-limb grip force was measured in Newtons (N) force and an average number of 10 trials were performed on each mouse to give a mean of hind-limb grip force which was normalized to body weight. Hind-limb grip force was investigated rather than fore-limb, as our behavioral observations and that of a previous study [12] noted only hind-limb dysfunction.

### Data analysis

Extracellular recording sites were analyzed if the frequency of EPCs and mEPCs did not change as a result of electrode pressure and if a single EPC was recorded within the first ten stimuli. Intracellular recordings were included if at least 100 EPPs and 30 mEPPs were recorded. Quantal content (*m*) was determined using method of failures [63] : *m* = log_e_ (number of stimuli/number of failures) utilizing intracellular electrophysiological recordings for diaphragm recordings (failures > 30%) or *m* = mean EPP amplitude/mean mEPP amplitude for EDL recordings. Binomial parameters were calculated from intracellular recording data using methods previously described [64]. The decay times, amplitude and frequency of evoked and spontaneous releases were calculated for each experimental group. Estimation of the readily releasable pool (RRP) size was conducted using a previously described method and model of assumptions [23, 24]. Unpaired Student's *t*-tests (two-tailed) were performed to compare mean values of WT and *lama*4^−/−^ mice at each age for electrophysiological, behavioral and morphological data unless otherwise specified. One way ANOVA with Tukey's multiple comparison tests were utilized to compare mean values of different age groups within the same genotype. Two way ANOVA with tukey's post hoc test was used to compare mean values between WT and mutant mice across 3MO and aged group for analysis of Bassoon density. Results were expressed as mean ± SEM with statistical significance accepted at *P* < 0.05.

## Abbreviations

[Ca^2+^]_O_: extracellular calcium concentration
α-BTX: alpha-bungarotoxin
AChR: Acetylcholine receptor
EDL: *Extensor Digitorum Longus*
EPCs: Endplate currents
EPPs: Endplate potentials
HFS: High frequency stimulation
*lama*4: Laminin alpha 4 gene
mEPCs: Miniature endplate currents
mEPPs: Miniature endplate potentials
NMJ: Neuromuscular junction
NTI: Nerve terminal impulse
PP: Paired-pulse
RMP: Resting membrane potential
RRP: Readily releasable pool
SNAP-25: Synaptosome-associated protein of 25 kDa
SV2: Synaptic vesicle protein 2
